# Safety and Efficacy of Lattice Radiotherapy in Voluminous Non-small Cell Lung Cancer

**DOI:** 10.7759/cureus.4263

**Published:** 2019-03-18

**Authors:** Beatriz E Amendola, Naipy C Perez, Xiaodong Wu, Marco A Amendola, Ian Z Qureshi

**Affiliations:** 1 Radiation Oncology, Innovative Cancer Institute, South Miami, USA; 2 Medical Physics, Innovative Cancer Institute, South Miami, USA; 3 Radiation Oncology, Innovative Cancer Institute, Cyberknife Center of Miami, South Miami, USA

**Keywords:** lattice radiotherapy, voluminous tumors, lung cancer, safety, efficacy

## Abstract

Objective

Lattice radiotherapy (LRT) is a novel technique of delivering heterogeneous doses of radiation to voluminous tumors not amenable to surgery. Built from the conventional two-dimensional grid, LRT utilizes the power of new technology, three-dimensional radiation allowing the delivery of higher doses of radiation to small spheres, also called vertices, inside bulky tumors while limiting exposure to surrounding healthy tissue. The main goals of the study were the evaluation of tumor response and the overall safety of LRT in this cohort of patients with bulky non-small cell lung cancer.

Materials and methods

During a seven-year period, 10 patients with non-small cell lung cancer (NSCLC), who presented with bulky, unresectable tumors, were treated using a single fraction of LRT followed by conventionally fractionated radiation. Patients received one initial LRT fraction of 18 Gy in the vertices and 3 Gy in the periphery. After the LRT, all patients continued with conventional radiation: 25 to 29 daily fractions of 1.8 Gy to 2 Gy.

Results

With a median follow-up of six months (range: one to 71 months), the mean decrease in tumor volume was 42%. The overall survival of the entire group ranged from four to 86 months (mean 22, median 16). There was no mortality related to LRT. No significant acute or chronic toxicity was noted.

Conclusion

In this small cohort, LRT appears to be a safe and effective modality to treat bulky NSCLC. Further research is needed to establish its efficacy in the management of voluminous NSCLC.

## Introduction

The management of voluminous lung tumors presents a challenge, particularly when surgical resection is not an option and conventional radiation and chemotherapy have limited efficacy for local control. An emerging technique is the use of lattice radiotherapy (LRT) [1]. Linear accelerator (LINAC)-based LRT can precisely deliver inhomogeneous high doses of radiation to different areas within the clinical target volume (CTV) while limiting the dose to the organs at risk adjacent to the tumor [2-3].

Recent case reports have demonstrated very effective local control of bulky lesions, thereby decreasing overall tumor burden and expanding options for subsequent surgical intervention [4-5].

High-dose 2D grid therapy has been available since the early 1900s but has not gained widespread acceptance by the radiation oncology community, presumably because of unacceptably high doses of radiation to normal tissues [6]. By adjusting the old 2D grid technique into a 3D lattice using multiple high-dose areas called vertices distributed within the central areas of the gross tumor volume (GTV), high-dose radiation is delivered within the bulk of the tumor and not in the peripheral areas adjacent to normal tissues. The term lattice, as described by Wu et al., has only a figurative purpose and does not imply a rigorous and symmetric repeated three-dimensional arrangement [1]. Instead, these vertices are placed inside the tumor, depending on its size and shape as well as the proximity of critical structures. The lattice technique may provide equivalent or superior clinical response in the management of large tumors while limiting toxicity to surrounding structures [4-5].

The common problems faced by the delivery of traditional radiation to a large tumor volume include poor blood supply and hypoxia within the tumor microenvironment, which stimulates factors such as hypoxia-inducible factor 2 alpha (HIF-2α), leading to protection against apoptosis. In addition, unacceptably toxic effects to the skin and surrounding tissues and difficulty in dosimetry for bulky lesions represent a challenge despite advances in intensity-modulated radiotherapy (IMRT) and image-guided radiotherapy (IGRT) [7]. These issues are largely mitigated by 3D dosimetry using multiple vertices within large solid lesions, and promising outcomes have already been seen in our early experience using these techniques [4-5].

There is a growing body of research emphasizing the modification of the tumor microenvironment following high-dose radiotherapy in bulky tumors, creating radiation-induced bystander effects (RIBE). These RIBEs can result in cell death to neighboring cancer cells where actual exposure to radiation is limited [7]. Apoptosis is attributed to the bystander effect thought to be secondary to cellular signaling either via direct physical contact in the case of gap junctures or by cell-released signaling molecules such as cytokines, nitric oxide, or reactive oxygen species [8-9]. The benefit of utilizing LRT would be the ability to destroy portions of the tumor receiving the highest dose of radiation while inducing RIBEs in those peripheral cells that are well within the target but farther away from healthy adjacent structures [7].

## Materials and methods

This is a retrospective review of the medical records for 10 patients treated with LRT with palliative intent. All patients were pathologically diagnosed with advanced NSCLC and they were not surgical candidates, having failed traditional systemic therapy with poor expected overall survival. None had received previous lung irradiation. There were nine males and one female; ages ranging from 49 to 87 years (mean 71, median 73). Five patients had stage III and five had stage IV lung cancers by virtue of distant metastases at presentation (Table 1). At the time of computed tomography (CT) simulation for radiotherapy (RT) planning, all patients had bulky lung tumors measuring greater than 5 cm in maximal diameter, with a range from 5 cm to 14 cm (mean 8 cm). Their initial gross tumor volume (GTV) ranged from 46 cc to 487 cc (mean 195 cc), defining the patients as having bulky lesions. The imaging follow-up period ranged from one month to 71 months (median six months). Nine of the 10 patients had a positron emission tomography (PET)-CT diagnostic study, which was used as a baseline assessment of disease burden.

**Table 1:** Patient characteristics at initial evaluation Abbreviations: C: cough; D: dyspnea; H: hemoptysis; P: pain; WL: weight loss; LRT: lattice radiotherapy; PET-CT: positron emission tomography-computed tomography; SUV: standardized uptake value; NSCLC: non-small cell lung cancer; NOS: not otherwise specified; LUL: left upper lobe (of lung); RUL: right upper lobe (of lung); RML: right middle lobe (of lung) * Greatest tumor diameter was greater than 5 cm according to planning CT.

Patient ID	G	Age	Stage	Location	Histology	KPS	Symptoms pre LRT	Tumor size in greatest dimension PET-CT	SUV PET-CT
1	M	72	IIIA - T3N1M0	LUL	Squamous	90	H, P, D	5.9 cm	15.5
2	M	87	IIIB - T4N2M0	RUL	Squamous	70	H, P, C	8.9 cm	23.9
3	F	74	IIIA - T2aN2M0	RML	NSCLC / NOS	70	D, C	6.7 cm	9
4	M	49	IV – T4N3M1b	LUL	Adeno	80	P, D	6.2cm, 3.9 cm	11.8, 9.2
5	M	87	IV – T4N2M1b	LUL	Adeno	70	WL, C	8.9 cm	10.42
6	M	62	IIIB – T4N3M0	LUL	Adeno	70	H, P	8.4 cm, 5.5 cm	19.75, 10.2
7	M	68	IV – T2AN0M1b	LUL	NSCLC / Neuro Endocrine	80	P, D	3.9 cm*	13.5
8	M	73	IV – T4N2M1b	RUL	Squamous and Adeno	70	WL, C, D	6 cm, 2.2 cm	11, 7.1
9	M	79	IIIA _ T3N2MX	LUL	Squamous	70	P	7.2 cm	N/A
10	M	55	IV – T3N1M1b	RUL	Adeno	80	P, C, D	5 cm	4.4

**Figure 1:**
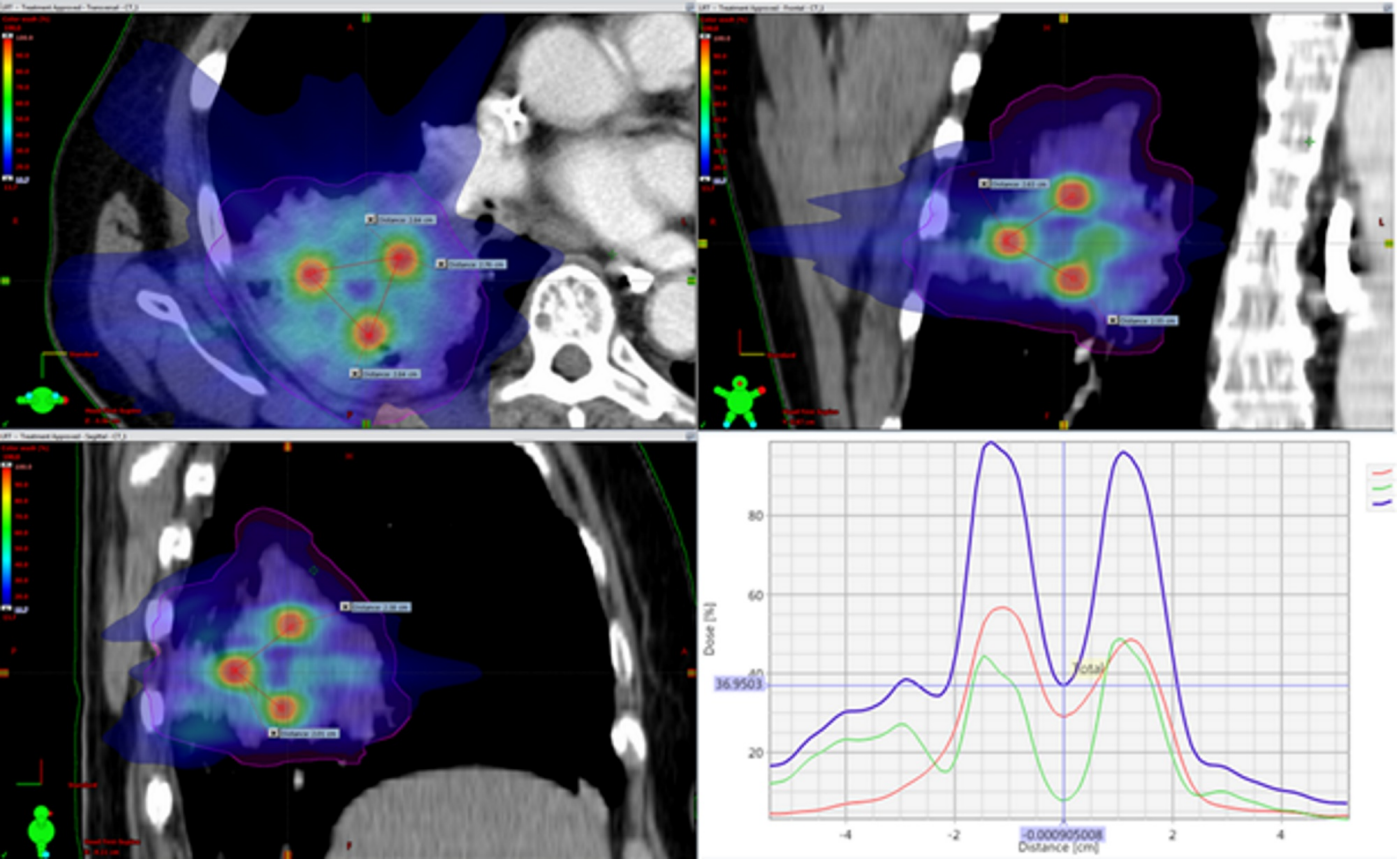
Representative scheme showing a lattice plan in one of our patients Representative scheme showing a lattice plan in one of our patients. The axial, coronal, and sagittal views are illustrating the percentage dose distribution in color wash. The red spheres represent the concentration of the highest dose in the three 1 cm vertices. The distribution graph demonstrates the separation between vertices, allowing for the dose to fall from 100% to 37%.

All patients were treated with one initial fraction of LRT with the lattice vertices receiving 18 Gy and the GTV receiving 3 Gy using 6 MeV photons (Figure 1). After LRT, all patients continued treatment with conventional fractionated external beam radiotherapy (EBRT) with 25 to 33 daily fractions of 1.8 Gy to 2 Gy. The number of vertices and its diameter depended on the size, shape, and location of the tumor with respect to the normal structures. The ratio of the dose between the vertices and the entire tumor volume ranged from 0.8% to 2.2%. The average distance between these high dose spheres (center to center) was 3.6 cm.

Seven patients received a boost or retreatment to the residual tumor. We considered a boost when the salvage, either radiosurgery or IMRT, was done within three months of the initial treatment. The term retreatment was used for those patients who remained with active disease beyond three months. The time from the end of the LRT plus conventional therapy to the time of the boost ranged from 20 days to nine months, with most patients receiving it within three months of LRT (Table 2). The volume of the lesions, characteristics of the vertices, and dose fractionation are shown in Table 2.

**Table 2:** Tumor and treatment characteristics Columns 1-7: Patient identifier, gross tumor volume GTV at the time of lattice radiotherapy (LRT) planning, corresponding equivalent sphere diameter, number, diameter, and volume of the vertices, ratio of the vertices’ volume and the GTV. Column 8-11: Radiation therapy after the LRT fraction showing first the conventional fractionated treatment, time between the conventional RT and the boost treatment, and the boost treatment dose using either RT (two patients) or stereotactic body radiation therapy (SBRT) (five patients). The total dose and the equivalent dose as given in 2 Gy/fx (EQD2) are also given in column 12.

Patient ID	GTV (cc)	Equivalent sphere diameter (cm)	# vertices	Vertice’s diameter (cm)	Vertices’ volume (cc)	Vertice’s volume / GTV (%)	Dose conventional [fxs x d/fx (Gy)]	% reduction last day of conventional	Time for boost or retreatment	CD	Total dose (Gy)		EQD2 (Gy)	
								(%)			GTV	VTV	GTV	VTV
1	218	7.5	3	1.5	4.75	2.2	29 x 2	83	0	0	61	76	61.25	100
2	239	7.7	7	0.8	2.55	0.9	25 x 1.8	19	2 months	3 x 8Gy	72	87	83.5	122.25
3	188	7.1	5	0.8	1.89	1	25 x 1.8	73	1 month	5 x 2.5Gy	60.5	75.5	60.52	99.27
4	146	6.5	3	1	1.2	0.8	25 x 1.8	55	1 month	3 x 7Gy	69	84	77	116
5	255	7.9	6	1	2.4	0.9	25 x 1.8	74	20 days 3 months	3 x 8Gy 5 x 5Gy	97	112	114.75	153.5
6	487	9.8	5	1.2	4.9	1	25 x 2	15	6 months	5 x 5 Gy	78	93	54.5	123.35
7	46	4.4	2	0.8	0.48	1	33 x 1.8	16	0	0	62.4	77.4	61.66	100.41
8	122	6.2	3	1.2	2.44	2	29 x 1.8	64	0	0	55.2	70.2	54.58	93.33
9	163	6.8	3	1.2	2.29	1.4	25 x 1.8	-70	9 months	7 x 3.5 Gy	72.5	87.5	75.06	113.81
10	110	6	3	1.2	2.2	2	29 x 1.8	67	3 months	5 x 8 Gy	95.2	110.2	114.58	153.33

Dose plans were created using Eclipse Planning System™ (Varian Medical Systems, Palo Alto, CA, USA). The volumetric arc (RapidArc) technique was used for treatment delivery with either the Varian Trilogy™ or Varian Edge™ linear accelerators (Varian Medical Systems, Palo Alto, CA, USA). A representative example of dosimetry and vertices placement can be seen in Figure 1.

**Figure 2:**
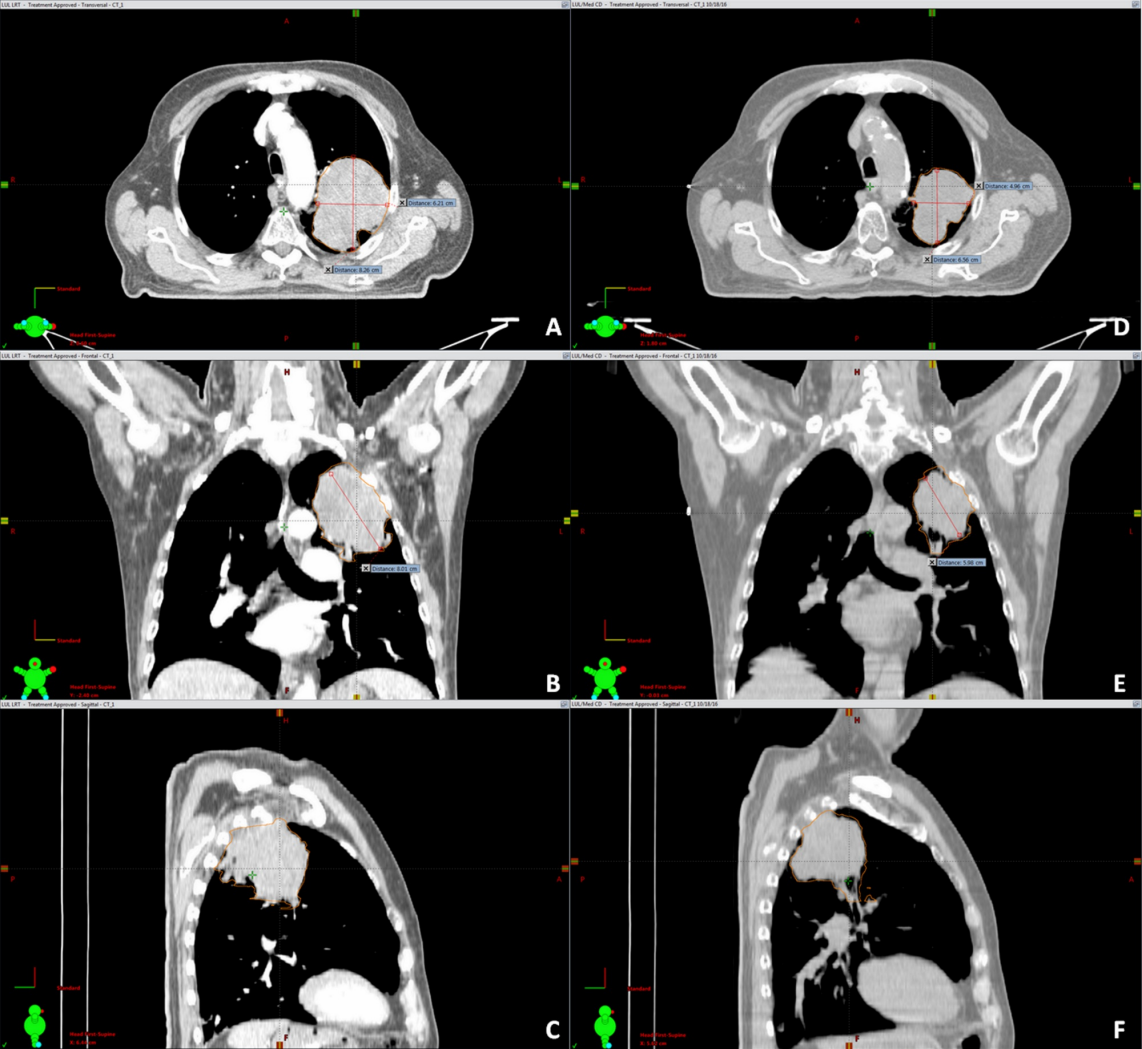
Significant decrease in tumor volume after completion of LRT followed by conventional XRT irradiation Significant decrease in volume after completion of lattice radiotherapy (LRT) followed by conventional irradiation in a 55-year-old male who presented initially with metastasis in the femur treated with radiation therapy. Following that, the primary non-small cell lung cancer (NSCLC) received LRT followed by conventional radiotherapy. After 18 months, the patient is alive with local control. Figures 2A, 2B, 2C (left): planning computed tomography (CT) scans in the axial (A), coronal (B) and sagittal views (C) before LRT with gross tumor volume (GTV) = 110.48 cc. Figures 2D, 2E, 2F (right): corresponding axial (D), coronal (E), and sagittal images (F) of the cone-beam CT (CBCT) obtained on the last day of treatment demonstrates a tumor reduction of 67% corresponding to a residual volume of 36.46 cc.

**Figure 3:**
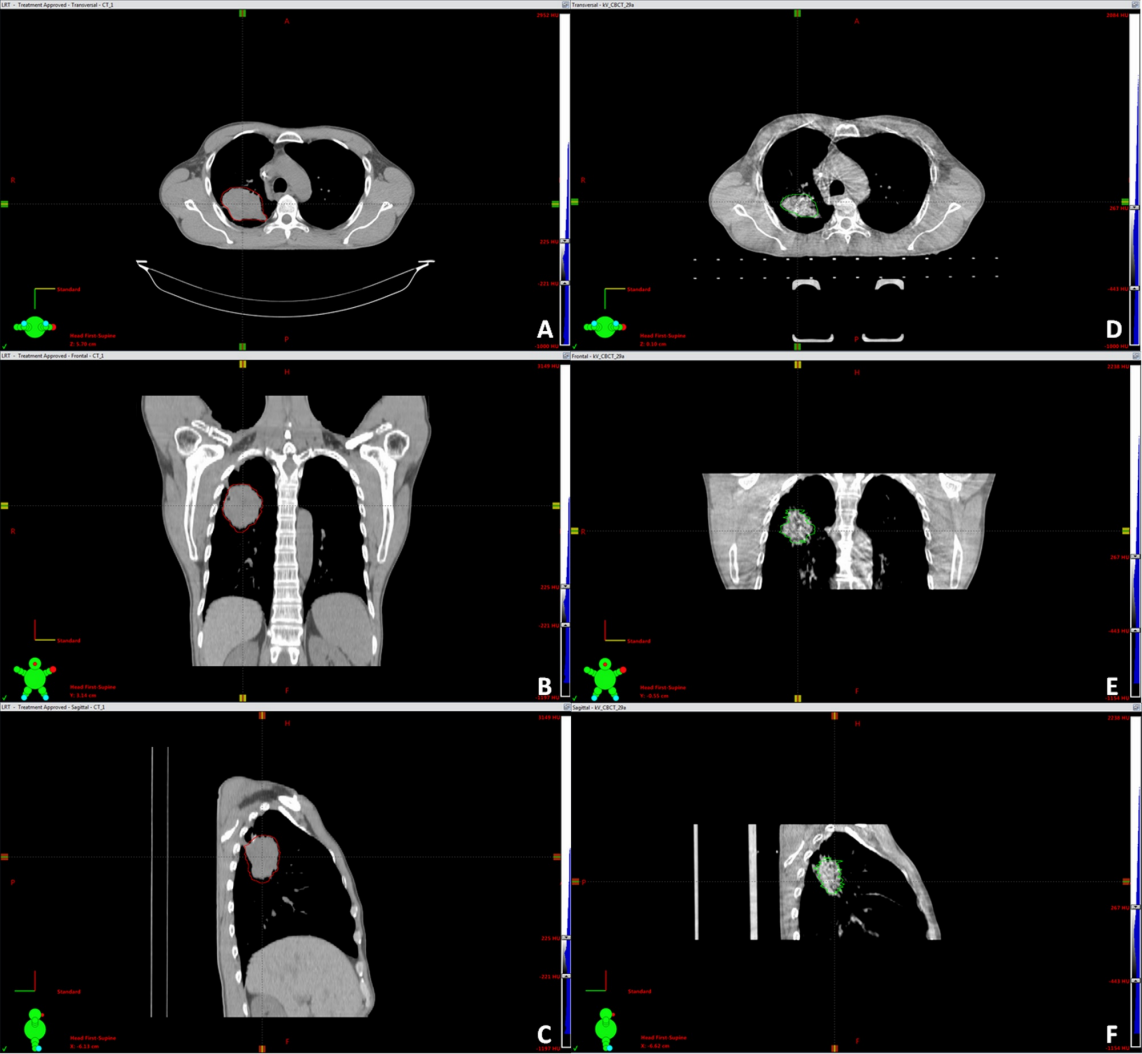
Adaptive therapy because of significant tumor response during treatment An 87-year-old male patient with non-small cell lung cancer (NSCLC) initially treated with GammaKnife (GK; Elekta Instrument AB, Stockholm) radiosurgery for brain metastases three months prior to lattice radiotherapy (LRT) and RT to the primary left upper lobe (LUL) tumor. The tumor volume decreased by 45%. Figures 3A, 3B, 3C: planning CT images in the axial (A), coronal (B), and sagittal views (C) before LRT with GTV = 255.55 cc. Figures 3D, 3E, 3F: New planning computed tomography (CT) images obtained in the axial (D), coronal (E), and sagittal views (F) for adaptive therapy planning necessitated after a significant decrease in volume to 139.6 cc after LRT and 18 conventional fractions. The patient is alive with the disease in a 24-month follow-up.

**Figure 4:**
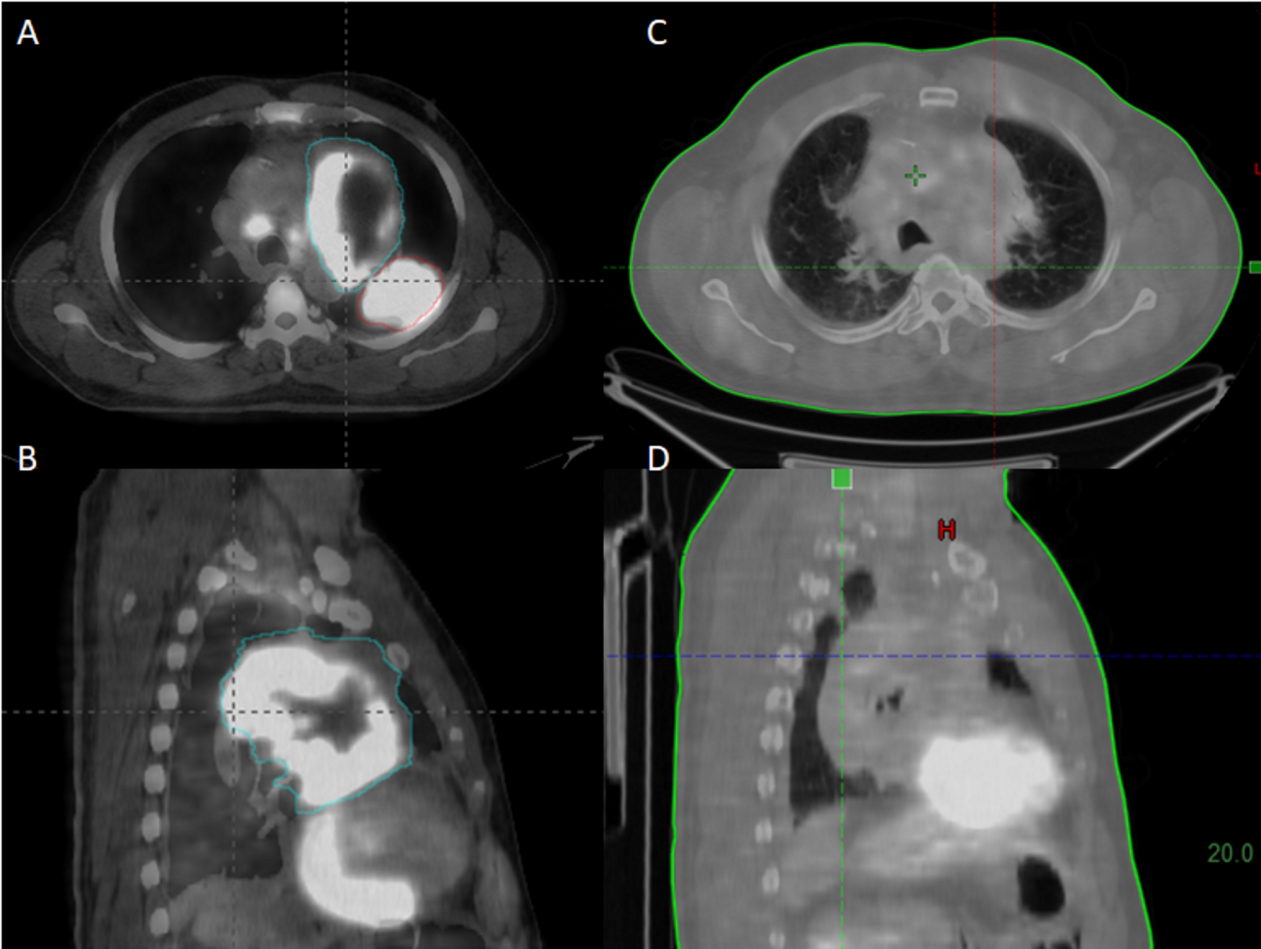
Complete response as per standardized uptake value (SUV) A 62-year-old male with advanced adenocarcinoma of the left lung. Figures 4A-4B: axial and sagittal views (soft tissue windows) of fluorodeoxyglucose (FDG) positron emission tomography-computed tomography (PET-CT) before LRT demonstrating two masses in the left lung active on PET and pre-tracheal adenopathy. Figures 4C-4D: corresponding axial and sagittal views (mediastinum windows) of follow-up FDG PET-CT 22 months after treatment showing a persistent mediastinal mass on corresponding CT but no significant uptake in the mediastinal mass or in adenopathy. The posterior mass in the left lung was no longer evident.

Image-guided radiation therapy (IGRT) was performed prior to every fraction using cone-beam CT (CBCT). Adaptive therapy was used for all patients when tumor response was rapid and significant, as seen in the daily CBCT (Figures 2-4).

The descriptions and grading scales found in the revised NCI Common Terminology Criteria for Adverse Events (CTCAE) version 4.0 were utilized for toxicity assessment [10].

Imaging response was evaluated in two ways: 1) by RECIST (Response Evaluation Criteria in Solid Tumors) criteria on post-treatment CT imaging, performed according to RECIST v 1.1 [11-13], and 2) by evaluation of FDG PET-CT scans [14] performed 12 weeks after the completion of treatment and repeated at least at every six-month interval. All scans were reviewed and compared to the baseline studies acquired prior to treatment. PET-CT response and its potential prognostic value are not completely understood in this setting; however, for the purpose of this study, we used a simplified form of the current standard, which is PERCIST: Positron Emission Tomography Response Criteria in Solid Tumors. PERCIST [15-16] defines four categories of treatment response:

· Complete metabolic response (CMR): A) decrease in average FDG uptake of all tumor lesions to the background and B) no new tumor lesions.

· Partial metabolic response (PMR): A) decrease in SUV activity of 30% or more from baseline and B) no new tumor lesions.

· Stable metabolic disease (SMD) - not CMR, PMR, or progressive metabolic disease (PMD).

· Progressive metabolic disease (PMD): A) increase in FDG activity of 30% or more from baseline measurements, B) increase in extent of FDG activity (bigger tumor), or C) new tumor lesions.

## Results

Tumor response

Tumor response from the day of initial CT simulation as compared to the cone-beam CT obtained on the last day of treatment demonstrated tumor volume reduction in nine out of 10 patients from 15% to 83% (median 64%, mean 52%). One patient, who had a large cavitated mass, experienced tumor growth in one week between the time of simulation and the beginning of treatment and did not respond while under treatment. Another patient died of intercurrent disease four months after completing the course of LRT and conventional radiation despite having demonstrated a reduction in tumor volume.

Tumor response was also evaluated from the day of initial CT simulation to the latest CT follow-up available, which ranged from one to 71 months (median six months). The tumor response based on the largest size reported in the follow-up study as compared to the initial CT, ranged from 10% to 53% (median 43%, average 37%), except for the same patient described above, where no response was noted after five months follow-up imaging.

At the time of the initial planning, the average size of the primary lesion was 7.7 cm (standard error of the mean: SEM 2.5 cm) with a decrease to an average of 5.6 cm (SEM 1.05 cm) at the end of the radiation treatment course, which was statistically significant with a p-value of 0.01.

Survival

Overall survival ranged from four to 86 months (median 16, mean 22). Five patients are alive, with a minimum of 18 months and a maximum of 86 months (median: 25 months). Three of them showed complete response with no activity as per the PET-CT scan despite having residual mass with a 48% average decrease in size on CT exam. The other two patients are alive with disease outside the chest and partial response of the treated tumor by PET-CT evaluation.

Toxicity

In all cases with imaging follow-up, there were changes on FDG-PET-CT suggestive of grade 1 radiation pneumonitis. There was no mortality attributable to LRT.

## Discussion

LRT was safely delivered to 10 patients with advanced stage NSCLC resulting in a statistically significant reduction in tumor size and in surprisingly long overall survival in patients otherwise deemed hospice candidates. This was accomplished without significant morbidity or mortality directly attributed to LRT.

An important limitation of this study is the small number of patients treated with LRT. This was secondary to the few patients who met the study criteria and were treated at our institution over a few years. A multicenter, prospective trial enrolling more patients with a standard protocol would be warranted based on the promising initial data presented here.

It would be intriguing to see if LRT induces immune responses to radiation, which could be clinically advantageous, especially at a time when immune therapy is generating high interest in clinical oncology. There are already some initial data suggesting that the tumor control seen by giving radiotherapy (RT) is, at least, in part, immune-mediated [17-18]. An investigation into a combination of RT with immune-directed therapy, such as nivolumab or pembrolizumab, is already ongoing [19].

Traditionally, it has been thought that radiation therapy was a local treatment modality without systemic effects. More recently, it has been found that there are biological alterations in non-irradiated cells caused by signals from irradiated cells close by within the irradiated volume. This has been called bystander effects. This is most likely caused by factors released from the treated cancer cells and from immune cells [20]. Radiation has been suggested to induce local inflammation and alter the T-cell pathways, resulting in cancer cell death. It has been suggested that the sizes of irradiated tumors may influence abscopal effects. Larger tumor tissues may be able to release more antigens in response to irradiation, which potentially intensifies abscopal effects than that from smaller size [20].

Eradicating a tumor requires total destruction of both tumor cells and its stromal contents. The experience of SRS and SBRT demonstrated that this is achievable for tumors of limited size, in terms of local control. Additionally, a high dose of radiation is shown to be immunogenic through the mechanism of “in-situ vaccine,” which triggers the cascade of antigen presentation toward the activation of CTL. Together with the secretion of factors such as IFN-γ, GM-CSF, TNF-α, IL-6, IL-10, or TGF-β, they can potentially mediate and contribute to the observable bystander effects and abscopal/systemic effects.

While effective in treating small tumors, SRS and SBRT are highly risky in treating voluminous tumors; the reasons being, first, a dose that is sufficiently high to eradicate both tumor cells and the whole stroma of a large tumor would likely be too toxic for the surrounding normal tissues and organs. Second, a complete, or near-complete coverage of the entire tumor, disrupting the total tumor vasculature and lymphatic drainage would prevent the circulation of “in-situ vaccine” and lymphocytes and, subsequently, diminish the immunogenic effects.

Applying LRT to a voluminous tumor would not incur additional toxicity to the surrounding normal tissues since the high-dose vertices (responsible for producing the “in-situ vaccine” and effective cytokines/chemokines) are confined within the tumor while preserving the vasculature and lymphatic drainage in the low-dose regions, allowing “immunogenic factors” to circulate and subsequently maximize bystander and abscopal effects.

Our results are in agreement with the recommendations of Feddock et al. who demonstrated that SBRT can be used safely to boost residual disease in advanced lung cancer [21]. Local regional control in locally advanced lung tumors is a well-known cause of failure. Escalating local therapy from conventional radiation using fusion with imaging, including PET-CT to select high-risk patients and using modern techniques (SBRT/lattice), allow for the intensification of treatment. We feel, as do Kalman et al., that a clinical trial incorporating this concept is highly indicated [22].

## Conclusions

In this retrospective review, early experience with LRT radiotherapy shows that it appears to be a safe and effective technique for delivering higher doses of cytotoxic radiation to bulky lung cancer lesions. As it has previously been hypothesized, bystander effects may be induced in peripheral neoplastic cells while avoiding toxicity to adjacent normal structures. LRT, by irradiating large areas from the center outward, provides a novel approach in the pursuit of modifying the tumor microenvironment while changing vascular access and drug delivery. It may allow for possible immune modulation of T-cells within the irradiated tissues, which is currently a trend in medical oncology and one that may prove to replace traditional chemotherapy as front-line therapy in certain tumor types.

Research could be expanded to other bulky primary malignancies, such as gynecologic tumors and sarcomas, where surgery may not be an option because of the anatomy involved or because of the patient’s comorbidities. Future studies using LRT are warranted based on the initial results of this study, showing safety and efficacy in utilizing this technique.
